# Food consumption patterns and nutrient intakes of infants and young children amidst the nutrition transition: the case of Lebanon

**DOI:** 10.1186/s12937-022-00779-9

**Published:** 2022-05-23

**Authors:** Lamis Jomaa, Nahla Hwalla, Fatima Al Zahraa Chokor, Farah Naja, Lynda O’Neill, Lara Nasreddine

**Affiliations:** 1 Department of Human Sciences, College of Health and Sciences, North Carolina Central University, Durham, NC 27707 USA; 2 Department of Nutrition and Food Sciences, Faculty of Agricultural and Food Sciences, American University of Beirut, Beirut, 11-0236 Lebanon; 3 Department of Nutrition and Dietetics, College of Health Sciences, University of Sharjah, 27272 Sharjah, UAE; 4 Nestlé Institute of Health Sciences, Nestlé Research, Société des Produits Nestlé S.A., 1015 Lausanne, Switzerland

**Keywords:** Food consumption patterns, Food groups, Nutrients, Macronutrients, Micronutrients, Intakes, Adherence, Under-five children, Lebanon

## Abstract

**Background:**

This is the first study on dietary intakes of infants and young children in the Eastern Mediterranean Region, a region that is currently witnessing the nutrition transition. It aims at characterizing food consumption patterns amongst 0–4 year old children in Lebanon, evaluating their macro- and micronutrient intakes and assessing adherence to dietary recommendations.

**Methods:**

Based on a national cross-sectional survey in 2012 (*n* = 866), the study collected data on sociodemographic and anthropometric characteristics, and one 24-hour dietary recall was administered. Nutrient intakes were compared with reference values: Estimated Average Requirement (EAR), Adequate Intake (AI) and Acceptable Macronutrient Distribution Range (AMDR).

**Results:**

Milk was the highest contributor to energy intake (EI) in infants (95.8 and 56.5% in 0–5.9 months and 6–11.9 months old infants, respectively), while its intake was lower among toddlers and preschoolers (35.4 and 15.1%, respectively). In contrast, intakes of sweets and sweetened beverages were the highest in preschoolers compared to younger children, contributing 18.5% EI in preschoolers. Compared to dietary guidelines, the lowest dietary adherence was found for vegetables (17.8–20.7%) and fruits (14.4–34.3%). Protein intake was within the recommendations for the vast majority of children. Although total fat intake was lower in toddlers and preschoolers compared to infants, more than 40% of toddlers and preschoolers exceeded the AMDR for fat and 87.3% of preschoolers exceeded the upper limit for saturated fat. Only 3.6% of toddlers and 11.5% of preschoolers exceeded the AI level for dietary fiber. Micronutrient intake assessment showed that mean intakes in infants exceeded the AI for all micronutrients, except for vitamin D and magnesium. In toddlers, vitamin D and calcium were below the EAR among 84.7, and 44.6%, respectively. In preschoolers, most of the children (91.9%) had inadequate intakes of vitamin D, and a third had inadequate intakes of folate, calcium and vitamin A.

**Conclusions:**

This study identified priority issues for nutrition intervention in infants and young children in Lebanon. Concerted multi-stakeholder efforts are needed to instill heathier food consumption and nutrient intake patterns early in life.

**Supplementary Information:**

The online version contains supplementary material available at 10.1186/s12937-022-00779-9.

## Background

Early life dietary practices play a critical role in fostering optimal growth and development in young children, and establishing long-term food preferences and dietary habits [1, 2]. Food consumption patterns early in life may also modulate the child’s future health trajectory and his/her susceptibility for the development of adult-onset chronic diseases [3, 4]. Despite the significance of dietary adequacy during this window of opportunity, information on dietary intakes amongst infants and young children remain scarce [5, 6]. This may be particularly true for the Eastern Mediterranean region (EMR), a region that is currently witnessing the nutrition transition, with its characteristic shifts in diet, lifestyle and body composition [7]. Young children may be amongst the most vulnerable population groups to the ongoing transition in lifestyle and dietary habits [8]. The modern food environment characterized by creative marketing and increased availability of high energy, low nutrient foods and beverages may adversely impact food consumption patterns amongst young children, potentially compromising their nutritional status [9]. In Lebanon, a small country of the Eastern Mediterranean basin, recent studies have reported increased adherence to the westernized dietary pattern amongst adults, adolescents and school-aged children, coupled with divergence away from the traditional Lebanese dietary pattern [10–12], which is recognized as a variant of the Mediterranean diet [13]. While little is known on how the nutrition transition is impacting the diets of infants and young children, there is evidence for an increase in the prevalence of overweight and obesity in this age group. It was for instance estimated that 9.1% of preschoolers were overweight or obese in 2012 [14], a value that exceeded the global prevalence estimate of preschool overweight/obesity (6.7%), as well as estimates reported from various developing countries (6.1%) [15]. In parallel, available evidence suggests that the burden of micronutrient deficiencies or inadequacies remains high amongst young Lebanese children, particularly for iron, zinc, calcium, folic acid and vitamin D [16]. This double burden of malnutrition in the pediatric population is of concern given its potential impact on growth, development, and disease susceptibility later in life.

Overcoming pediatric malnutrition, in all of its forms, necessitates the development of evidence-based interventions and policies to ensure the availability of and access to healthy diets [16]. Successful planning for such interventions ought to be guided by comprehensive and accurate data on dietary intakes amongst children. Previous studies investigating dietary intakes and food consumption patterns amongst young Lebanese children are lacking, with only sporadic evidence suggesting inadequate or suboptimal intakes of some specific nutrients [16]. It is in this context that this study was conducted, with the aim of 1) characterizing food consumption patterns amongst Lebanese infants and young children, and shedding light on how these consumption patterns may vary between subgroups of different ages; 2) assessing adherence of Lebanese children to dietary recommendations; and 3) evaluating their macro- and micronutrient intakes in comparison with the US dietary reference intake values, given that there are no reference values specifically for the local population. The study data were based on the national survey “Early Life Nutrition and Health in Lebanon” (ELNAHL), which is an individual food consumption survey that was conducted in 2012 on a nationally representative sample of Lebanese infants and young children aged less than 5 years [17]. The data analysis protocol was based on the one adopted by the “Feeding Infants and Toddler Study” (FITS), which is a dietary intake survey of large cross-sectional samples of infants and toddlers (0–4 years) that was conducted in 10 different countries, using consistent methodologies, to allow for comparisons and nutrition knowledge sharing [18, 19]. The study findings will contribute to a better understanding of food consumption patterns in children, and hence allow for a prioritization of policies and interventions aimed at developing healthy eating habits early in life.

## Methods

### Study population

This is a cross-sectional study based on the national survey, “Early Life Nutrition and Health in Lebanon, ELNAHL”, conducted in Lebanon in 2012 [14, 20]. The survey comprised a representative sample of Lebanese children (0–5 years) and their mothers. Details regarding the sampling are published elsewhere [14, 20]. In brief, households were considered as the primary sampling units in this survey. The selection of households was based on a stratified cluster sampling strategy, with the strata being the six Lebanese governorates and the clusters being selected further at the level of districts. In each district, the selection of households was performed according to a probability proportional to size approach, whereby a higher number of participating households was drawn from more populous districts; the selection of households was carried out using systematic sampling. To be eligible to participate in the survey, households had to include a mother and a child aged 5 years or less. Of the 1194 eligible households that were contacted, 1029 agreed to participate in the survey, with a response rate of 86%. Children and their mothers were excluded from the study if children were of non-Lebanese nationality, born preterm (< 37 weeks), or had any chronic disease, inborn error of metabolism, or physical malformation that could alter dietary intake or body composition [14]. Children who were reported by their mother as being ill during the past 24 hours (i.e. on the day that would be covered during the dietary intake) were also excluded from the study.

For the present study, data pertinent to infants, toddlers, and preschoolers aged between 0 and 4 years were considered (*n* = 866), in line with the FITS protocol that was adopted in reporting and analysis [18, 19]. In brief, FITS uses 24 hour dietary recalls to assess individual-level food consumption and dietary intakes for children from birth till 4 years of age [18, 19]. It further stratifies infant and young children into 4 different age groups, as follows: 0–5.9 months; 6–11.9 months; 12–23.9 months; and 24–47.9 months.

### Data collection

Data collection was performed in the household setting through face to face interviews with the mothers. Trained research nutritionists conducted the data collection, using an age-specific multi-component questionnaire [14]. Information about socio-demographic and lifestyle characteristics of the study participants was obtained, and comprised sex of the child, household monthly income in Lebanese pounds, crowding index (based on number of rooms and number of individuals living in the household), mother’s and father’s education levels and employment status, and marital status of the mother.

The study was performed according to the guidelines specified by the Declaration of Helsinki and the study protocol was approved by the Institutional Research Board, American University of Beirut (Protocol number NUT.LN.13). Written informed consent was obtained from all participating mothers prior to enrollment in the study.

### Anthropometric assessment

Anthropometric characteristics including weight and height were measured for all participating children. Length was measured amongst children ≤2 years old without clothes and diapers using an infantometer to the nearest 0.1 cm (SECA 210); weight was measured on an electronic pediatric scale to the nearest 0.1 kg (SECA 354). For children aged above 2 years, height measurements were obtained without shoes, using a stadiometer, and body weight was measured to the nearest 0.1 kg with the participant in light indoor clothing and with bare feet or stockings, using a standard clinical scale (Seca 11,770). All measurements of weight and height were taken twice, and the average values were adopted. Body mass index (BMI) was calculated as the ratio of weight (kilograms) to the square of height (meters) [21]. Anthropometric measurements were interpreted using the World Health Organization (WHO)-2006 criteria based on sex and age specific z-scores [22].

### Dietary intake of children

Trained nutritionists performed the dietary intake assessment using the United States Department of Agriculture (USDA) Multiple Pass 24- Hour Dietary Recall (24-HR) approach, with mothers acting as proxies [23]. In the case where another caretaker shared the responsibility of child feeding, the mother consulted directly with him/her for additional information/clarification pertinent to the child’s dietary intake. The specific steps that were adopted during the dietary interview included: 1) quick food list recall, 2) forgotten food list probe, 3) time and occasion at which foods were consumed, 4) detailed overall cycle and 5) final probe review of the foods consumed.

Food items, as consumed, were categorized into 10 food groups based on similarity in nutrient profile and culinary use. The food groups included Grain and grain products; Fruits; Vegetables; Milk and milk products; Meats and other protein sources; Mixed dishes; Savory snacks; Sweets, sweetened beverages, and desserts; Fats and oils; and Condiment and sauces.

To allow for comparison with the American Heart Association/American Academy of Pediatrics (AHA/AAP) recommendations for healthy eating patterns in children [24, 25], all recipes were disaggregated into their individual ingredients. Individual food items were then grouped into five main groups (Milk/Dairy; Lean Meats/Beans; Fruits; Vegetables; Grains), and intakes of these food groups were compared with the AHA/AAP recommended number of servings from each food group (by gender and age) [24, 25]. Because dietary guidelines are not yet developed for children under 1 year of age, it was not possible to assess dietary adherence for this group of children; hence our adherence analysis was restricted to children aged 12–23.9 months and 24–47.9 months.

Dietary intake data were analyzed using the Nutritionist Pro software (version 5.1.0, 2014, First Data Bank, Nutritionist Pro, Axxya Systems, San Bruno, CA). Within the Nutritionist Pro software, the USDA database was selected. For composite, mixed, and traditional dishes, standardized recipes were added to the Nutritionist Pro software using single food items. Daily energy, macro-and micronutrient intakes were estimated for each participating child. Energy and nutrient intake from breastmilk was also assessed using the Nutritionist Pro software. On the interview day, 148 children (17.1%) had consumed breast milk on the day (63.8% of 0–5.9 months old infants; 38.2% of 6–11.9 months old infants and 10.7% of toddlers aged 12–23.9 months). Thus, during the 24-HR, the mother reported the number of times and the duration of breastfeeding. To determine the amount of human milk consumed and the corresponding nutrient intake provided, we utilized the methodology previously described in the FITS [26]. Participants who were exclusively breastfed were allocated a standard reference value of 780 mL/day of human milk if aged 0–5.9 months and 600 mL/day of human milk if aged 6–11.9 months. If the participant was partially breastfed the amount of human milk allocated was 780 mL/day minus the total amount of “formula milk (mL/day)” consumed on the day of the recall if aged 0–5.9 months; or 600 mL/day minus the total amount of “formula milk (mL/day)” consumed if aged 6–11.9 months. In children aged 12–17.9 months and 18–23.9 months, the total daily amount of human milk was calculated as 89 mL or 59 mL, respectively, for every reported feeding occasion.

Dietary energy intake (EI) was compared with the child’s estimated energy requirements (EER). EER for each child were calculated using the 2006 Institute of Medicine of the National Academies published Guide [27]. The equations are sex and age specific. For children aged less than 3 years, EER equations do not account for physical activity level. For children aged above 3 years, EER equations account for four levels of physical activity (PAL); sedentary (PAL 1.0–1.39), low activity (PAL 1.4–1.59), active (PAL 1.6–1.89) and very active (PAL 1.9–2.5). In our study, data collection did not obtain information on physical activity or sedentary behavior. Accordingly, for children aged 3 to 4 years, low physical activity (PA coefficient = 1.13 for boys and 1.16 for girls) was adopted in EER calculations.

### Statistical analysis

All analyses were stratified by age, in line with the FITS protocol [18]. Accordingly, the sample was divided into 4 age groups: 0–5.9 months; 6–11.9 months; 12–23.9 months; and 24–47.9 months.

Sociodemographic characteristics were described using frequencies and percentages as well as means and standard errors (SE) of means for categorical and continuous variables, respectively. Anthropometric characteristics were interpreted using the WHO-2006 criteria for under-five year old children [22]. Food sources of energy (Kcal/capita/day) and percent contribution to EI (%EI) were calculated. Percentage adhering to the AHA/AAP dietary recommendations were assessed and differences between age groups were evaluated using chi-square test and Fisher’s exact test, as applicable.

Macro and micronutrient intakes were reported as means and SE of means for the four age groups, and percentage of kilocalories from a given macronutrient was calculated. Estimated nutrient intakes were compared to age-specific Dietary Reference Intakes (DRIs) established by the Institute of Medicine (IOM), including the Estimated Average Requirement (EAR), Adequate Intake (AI) [28], and the Acceptable Macronutrient Distribution Range (AMDR). Sodium and Potassium DRIs were based on the updated National Academies recommendations [29]. For nutrients with an EAR, the proportions of children with intakes less than the EAR (i.e. inadequate intakes) was calculated by age group. AI was used for nutrients that do not have an EAR value and the percentage of children consuming greater than or equal to the AI was calculated. AI is “a recommended average daily nutrient intake level based on observed or experimentally determined approximations or estimates of nutrient intake by a group (or groups) of apparently healthy people that are assumed to be adequate [30].” Thus, a group mean intake at or above the AI indicates that the prevalence of inadequacy is probably low [30]. If a group’s mean intake is below the AI, then intakes may need to increase, but it is not possible to precisely quantify the prevalence of inadequacy [30]. The proportions of children with intakes outside the upper or lower bounds of the AMDR for fat, protein and carbohydrate were examined for children older than 1 year of age. Statistical analyses were performed using STATA (version 12.0), and differences were considered statistically significant at *P* < 0.05.

## Results

### Sociodemographic and anthropometric characteristics

Table 1 shows the sociodemographic characteristics of the study sample, which consisted of 866 children aged between 0 to 3.9 years. Almost half of the children (45.4%) were aged between 24 to 47.9 months. Within this age category, the majority were boys (52.7%) and from North Lebanon (33.8%). Only 16.9% of children across the 4 age groups had a monthly family income greater than 2,000,000 LBP (equivalent to 1333 USD). Similarly, only 13.7% of the households had a crowding index below 1 (indicating a higher socioeconomic status) [31]. The majority of children’s mothers and fathers in the 4 age groups had an educational level ranging between elementary and secondary school. While almost all of the children’s fathers were employed (percentages ranging from 94 to 98% across the age groups), a substantial proportion of mothers did not work at the time of the survey (percentage ranging from 75 to 86% across the age groups). The vast majority of participating mothers were married at the time of the interview (98.8%).

**Table 1:** Socio-demographic and anthropometric characteristics of Lebanese infants and young children, by age

Age group (months)	Total		0–5.9		6–11.9		12–23.9		24–47.9		***p***-value*
Sociodemographic Characteristics	n	%	n	%	n	%	n	%	n	%	
	866	100	103	11.9	148	17.1	222	25.6	393	45.4	
**Sex**											
**Male**	440	50.8	53	51.5	65	43.9	115	51.8	207	52.7	0.326
**Female**	426	49.2	50	48.5	83	56.1	107	48.2	186	47.3	
**Governorate**											
**Beirut**	80	9.2	12	11.6	14	9.5	11	4.9	43	10.9	0.149
**Mount Lebanon**	264	30.5	30	29.1	41	27.7	69	31.1	124	31.5	
**North Lebanon**	319	36.8	35	34.0	68	45.9	83	37.4	133	33.8	
**South and Nabatieh**	136	15.6	19	18.4	18	12.2	36	16.2	63	16.0	
**Bekaa**	67	7.8	7	6.8	7	4.7	23	10.4	30	7.6	
**Household monthly income (Lebanese pounds)**^**a**^											
**< 1,000,000**	286	33.0	27	26.2	64	43.2	76	34.2	119	30.3	0.001
**1,000,000-2,000,000**	271	31.3	44	42.7	30	20.3	61	27.5	136	34.6	
**> 2,000,000**	146	16.9	17	16.5	27	18.2	29	13.1	73	18.6	
**Do not know / Refused to answer**	163	18.8	15	14.6	27	18.2	56	25.2	65	16.5	
**Crowding Index**											
**< 1 persons/room**	119	13.7	16	15.5	26	17.6	30	13.5	47	12.0	0.366
**≥ 1 persons/room**	747	86.3	87	84.5	122	82.4	192	86.5	346	88.0	
**Education of mother**											
**Less than elementary**^**b**^	25	2.9	1	1.0	5	3.4	6	2.7	13	3.3	0.328
**Elementary-secondary**^**c**^	653	75.4	72	69.9	107	72.3	169	76.1	305	77.6	
**College**	188	21.7	30	29.1	36	24.3	47	21.2	75	19.1	
**Education of father**											
**Less than elementary**^**b**^	33	3.9	3	2.9	7	4.8	13	5.9	10	2.6	0.532
**Elementary-secondary**^**c**^	694	81.3	82	80.4	118	80.3	174	79.4	320	82.9	
**College**	127	14.9	17	16.7	22	15.0	32	14.6	56	14.5	
**Mother employed**											
**Yes**	150	17.3	25	24.3	25	16.9	45	20.3	55	14.0	0.049
**No**	716	82.7	78	75.7	123	83.1	177	79.7	338	86.0	
**Father employed**											
**Yes**	822	95.7	99	96.1	145	98.0	209	94.6	369	95.4	0.440
**No**	37	4.3	4	3.9	3	2.0	12	5.4	18	4.6	
**Marital status**											
**Married**	856	98.8	103	100.0	148	100.0	221	99.6	384	97.7	0.063
**Separated, divorced, or widowed**	10	1.2	0	0.0	0	0.0	1	0.4	9	2.3	
**Anthropometric Characteristics**											
**Height for age**^**d**^											
**Stunted**	61	7.1	7	6.8	7	4.7	22	10.1	25	6.4	0.219
**Not stunted**	798	92.9	96	93.2	141	95.3	197	89.9	364	93.6	
**BMI Status**^**e**^											
**Wasted**	17	2.0	4	3.9	8	5.4	4	1.8	1	0.3	< 0.001
**Normal**	511	59.5	69	67.0	98	66.2	106	48.4	238	61.2	
**At risk of overweight**	223	25.9	13	12.6	33	22.3	67	30.6	110	28.3	
**Overweight**	79	9.2	11	10.7	7	4.7	33	15.1	28	7.2	
**Obese**	29	3.4	6	5.8	2	1.3	9	4.1	12	3.1	

*Abbreviations*: *BMI* body mass index, *HAZ* height-for-age z-score, *n* sample size, *US* United States, *WHO* World Health Organization

^*^Chi–square test was used for all variables in the table, except for marital status where Fisher’s exact test was used

^a^1 US dollars = 1, 500 Lebanese pounds

^b^ Less than elementary includes being illiterate, not attending school, or being able to read and write only

^c^ Elementary to secondary includes primary school, intermediate school, high school, or technical diploma

^d^ Stunted if HAZ < -2, not stunted if HAZ ≥ -2

^e^ Anthropometric measurements of children were categorized based on WHO classification

The anthropometric characteristics of the study sample are also shown in Table 1. The prevalence of stunting was estimated at 7.1% in the total sample, with no significant differences between age groups. BMI status differed significantly between age groups. The prevalence of wasting was 2% in the overall study sample, with the highest levels being observed in the 6–11.9 months age group (5.4%). Approximately a quarter of participating children (25.9%) were at risk of overweight, with the prevalence increasing with age, while 9.2 and 3.4% were overweight and obese, respectively.

### Energy intakes

Dietary EI estimations are shown in Table 2. EI increased with age, from 608 kcal/day in 0–5.9 months old infants to 1504 kcal/day in 24–47.9 months old children. Dietary EI values were compared to the EER: percent EER varied between 124.2 and 132.8%. The proportions of children consuming energy below the EER ranged between 25.2 and 34.5%, while the proportions of those exceeding the EER ranged between 65.5 and 74.8% across age groups. No significant differences were noted between the different age categories.

**Table 2:** Estimated dietary energy intake amongst Lebanese infants and young children, and percent contribution to estimated energy needs, by age

	Age group			
	0–5.9 months	6–11.9 months	12–23.9 months	24–47.9 months
	**Mean ± SE**			
**Energy intake (kcal)**	608.0 ± 14.2	899.1 ± 28.2	1205.6 ± 33.0	1504.1 ± 27.0
**EER**	498.6 ± 15.1	730.5 ± 8.7	934.0 ± 10.7	1232.2 ± 9.2
**% EER**	132.8 ± 4.9	124.2 ± 3.8	132.1 ± 4.0	124.2 ± 2.4
	**Energy inadequacy n (%)**			
**% below EER**	26 (25.2)	51 (34.5)	70 (31.5)	125 (31.8)
**% above EER**	77 (74.8)	97 (65.5)	152 (68.5)	268 (68.2)

%EER = Energy Intake/EER × 100

No significant differences in proportions of children below or above EER between age groups (p-value = 0.478)

*Abbreviations*: *EER* Estimated energy requirement, *SE* standard error

### Food consumption patterns

**Fig. 1:**
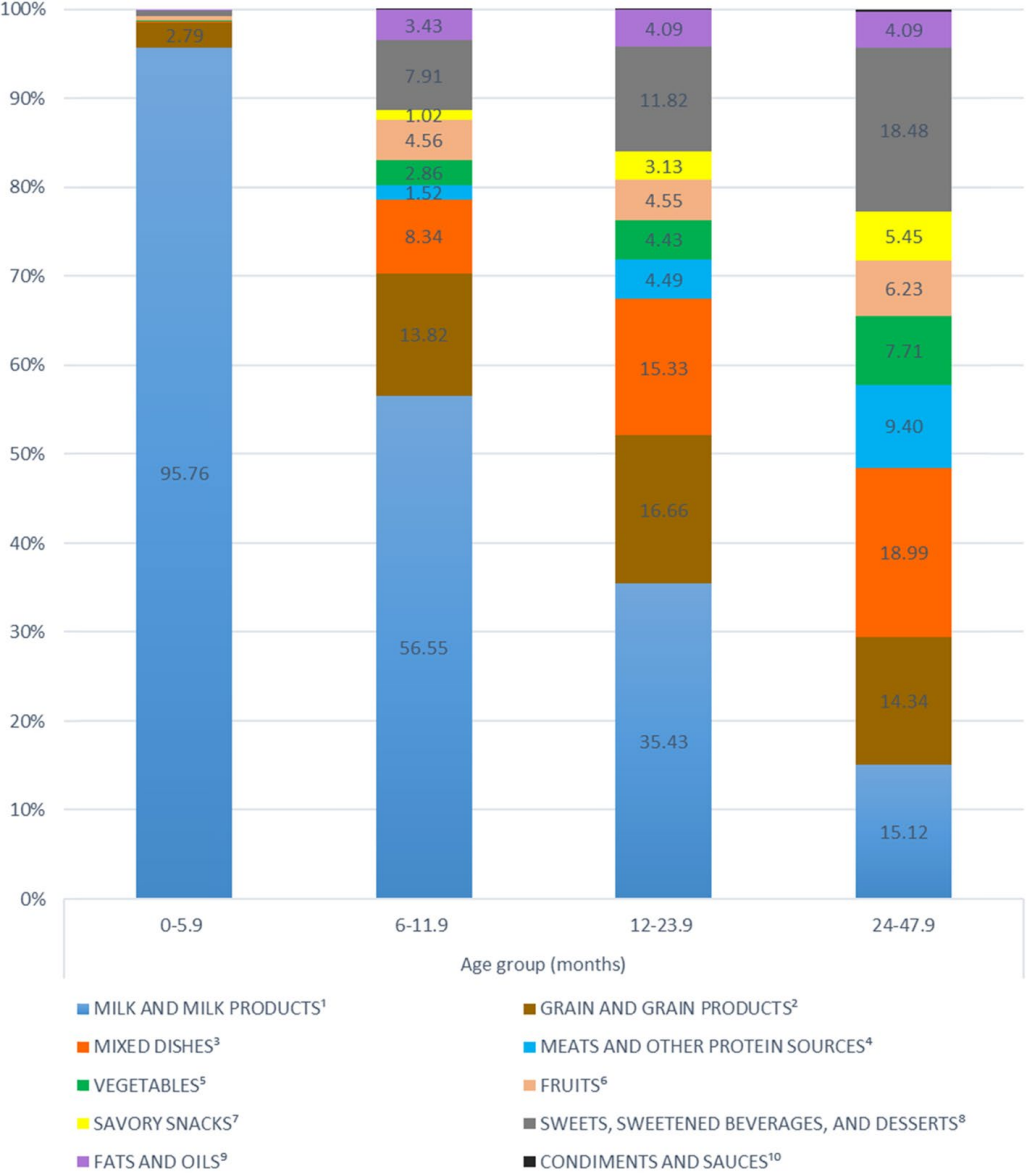
Percent of energy from major food groups, as consumed, in 0–48 months Lebanese children. Legend: ^1^ Includes any milk (breast milk, infant formula, cow’s milk, and goat’s milk) as well as dairy foods, cheeses, and yogurt. ^2^ Includes bread, rolls, pita, saj, baby food cereals/grains, baby food finger food, cereals, crackers, pretzels, kaak, pancakes, French toast, pasta, rice, and other grains. ^3^ Includes all yogurt, grain, and meat based mixed dishes such as sandwiches, macaroni and cheese, spaghetti and lasagna, sandwiches, beans and rice, pizzas, Mahashi, and soups. ^4^ Includes any baby food and non-baby food meats, dried beans, peas and legumes, eggs, peanut butter, nuts and seeds. ^5^Includes baby food vegetables, canned, cooked and raw vegetables, white potatoes, and 100% vegetable juice. ^6^ Includes baby food fruits, canned, dried, and raw fruits, 100% baby food juices, and other 100% fruit juices. ^7^ Includes popcorn, potato chips, and corn chips. ^8^ Includes baby food desserts and cookies, non-baby food dessert items (cakes, pies, cookies, bars, brownies, biscuits, pastries, muffins, and traditional desserts), ice cream and dairy desserts, puddings, candy, cereal and nutrition bars, gelatins, ices, and sorbets, sugars, syrups, preserves, and jelly, fruit drinks and other sugar sweetened beverages. ^9^Includes butter, margarine, animal fats, dressings, oils, and olives. ^10^Includes condiments, herbs, seasonings, gravies, and sauces

Food items, as consumed, were categorized into 10 food groups. Figure 1 shows the intake of these food groups, expressed as percent of EI (Table A1 in the additional material shows the intakes of these food groups in g/day). Early in life, the major source of dietary energy came from milk, contributing 95.8% of energy in 0–5.9 months old infants (Fig. 1). A small amount of other food groups was introduced, with 2.8% of energy from grains and very little from other food groups. By 6 months, all food groups have been introduced into the diet of infants, but milk and milk products remained the main source of dietary energy (56.5% EI) in 6–11.9 months old infants. The other most important contributor to EI included grains (13.8% EI) and mixed dishes (8.3% EI) in this age group. Diversification of the diet continued to increase with age, paralleled by a decrease in the intake of milk. In toddlers aged 12–23.9 months, milk and milk products contributed 35.4% of EI, followed by grain and grain products (16.7%), mixed dishes (15.3%) and sweets, sweetened beverages and desserts (11.8%). In children aged 24–47.9 months, the main contributors to EI were mixed dishes (18.9%), followed by sweets, sweetened beverages and desserts (18.5%), milk and milk products (15.1%) and grains and grain products (14.3%).

### Adherence to dietary recommendations

**Fig. 2:**
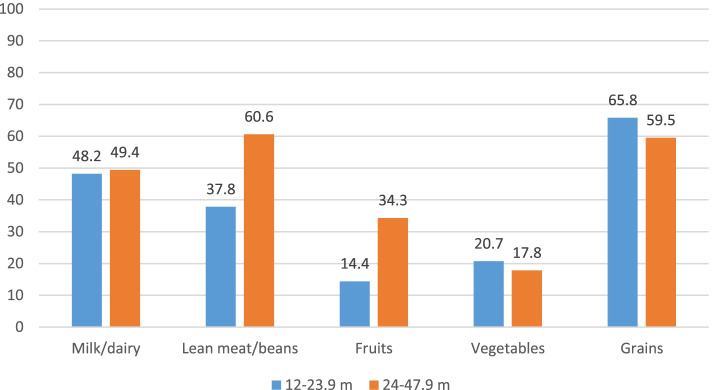
Adherence to dietary recommendations pertinent to food group intake^#^, in children aged above 1 year. Legend: Adherence assessment was based on the recommended servings for the various food groups by age and gender based on the AHA/AAP Dietary Recommendations for Children [24, 25]**.** ^**#**^ Recipes of composite foods were disaggregated prior to the assessment of adherence to dietary recommendations. * Indicates significant difference between the age groups in the proportion of children adhering to food group recommendations. Abbreviations: AHA/AAP: American Heart Association/American Academy of Pediatrics

In children aged above 1 year, the intake level of the different food groups was compared to dietary recommendations set by AHA/AAP [24, 25], after the disaggregation of composite dishes (Table A2 in the additional material shows the intakes of these food groups in g/day). In these children, the highest adherence was observed for the grains food group (59.5–65.8%), while the lowest adherence was found for vegetables (17.8–20.7%) and fruits (14.4–34.3%). Only half of the children in 12–23.9 months and 24–47.9 months age groups were adherent to milk and dairy (48.2% vs. 49.4%, respectively). Significant differences in adherence to fruit intake recommendations were noted between the two age groups: 34.3% of children aged 24–47.9 months were adherent, compared to only 14.4% of younger children aged 12–23.9 months. Similar observations were noted for the lean meat/beans food group (60.6% vs. 37.8%) (Fig. 2).

### Macronutrient intakes

Macronutrient intakes (absolute and %EI) are displayed in Table 3 by age group. The estimated macronutrient intakes in absolute terms and as a percentage of daily EI reflect the predominantly milk-based diets during infancy. Fat intake contributed almost half of EI in infants under 6 months of age and approximately 43% EI in infants aged 6 to 11.9 months. The majority of infants (83.5 and 77% of 0–5.9 months old and 6–11.9 months old infants, respectively) exceeded the AI for total fat. In contrast, only 38.8% of 0–5.9 months old infants had carbohydrates intakes above the AI level. This proportion increased to 52.1% in those aged 6–11.9 months. The majority of 0–5.9 months old infants (68%) exceeded the AI for protein, while only 6.1% of 6–11.9 months old infants had inadequate protein intakes (<EAR).

**Table 3:** Macronutrient intakes amongst Lebanese infants and young children, by age

Age group	0–5.9 months			6–11.9 months				12–23.9 months					24–47.9 months				
Nutrient	DRI	Mean ± SE	% > AI	DRI^a^	Mean ± SE	% < EAR	% > AI	DRI^a^	Mean ± SE	% < EAR or <AMDR	% > AI	% > AMDR	DRI^a^	Mean ± SE	% < EAR or < AMDR	% > AI	% > AMDR
	AI			AI/EAR				AI/EAR/AMDR					AI/EAR/AMDR				
**Macronutrients**																	
Energy (kcal/d)	–	608.0 ± 14.2	–	–	899.1 ± 28.4	–	–	–	1205.6 ± 33.0	–	–	–	–	1504.1 ± 27.0	–	–	–
Total Fat (g/d)	31	34.7 ± 0.8	83.5	30	42.1 ± 1.3	–	77.0	–	53.1 ± 1.6	–	–	–	–	65.7 ± 1.5	–	–	–
Saturated fat (g/d)	–	13.9 ± 0.3	–	–	14.2 ± 0.5	–	–	–	15.7 ± 0.6	–	–	–	–	21.9 ± 0.6	–	–	–
Monounsaturated fat(g/d)	–	12.8 ± 0.3	–	–	13.7 ± 0.5	–	–	–	17.7 ± 0.6	–	–	–	–	23.0 ± 0.6	–	–	–
Polyunsaturated fat(g/d)	–	5.7 ± 0.2	–	–	8.3 ± 0.4	–	–	–	11.8 ± 0.5	–	–	–	–	12.7 ± 0.4	–	–	–
Linoleic acid (g/d)	4.4	4.8 ± 0.2	47.6	4.6	7.6 ± 0.4	–	72.3	7	11.1 ± 0.5	–	68.5	–	7	12.0 ± 0.4	–	70.0	–
Alpha linolenic acid (g/d)	0.5	0.6 ± 0.0	48.5	0.5	0.7 ± 0.0	–	62.2	0.7	0.8 ± 0.0	–	53.6	–	0.7	0.8 ± 0.0	–	48.6	–
Carbohydrate (g/d)	60	64.4 ± 1.8	38.8	95	110.2 ± 3.9	–	52.1	**100**	150.0 ± 4.2	21.2	–	–	**100**	182.2 ± 3.5	11.4	–	–
Total Sugar (g/d)	–	60.7 ± 1.5	–	–	69.7 ± 2.4	–	–	**–**	74.9 ± 2.2	–	–	–	–	76.8 ± 1.8	–	–	–
Added sugar (g/d)	–	0.9 ± 0.3	–	–	10.6 ± 1.4	–	–	**–**	19.5 ± 1.5	–	–	–	–	34.1 ± 1.6	–	–	–
Protein (g/d)	1.52 g/Kg	10.9 ± 0.3	68	**1 g/Kg/d**	21.7 ± 0.9	6.1	–	**0.87 g/Kg**	34.8 ± 1.2	1.8	–	–	**0.87 g/Kg**	50.9 ± 1.1	1.3	–	–
Dietary fiber (g/d)	–	0.2 ± 0.1	–	–	4.0 ± 0.3	–	–	19	7.7 ± 0.4	–	3.6	–	19	11.1 ± 0.3	–	11.5	–
**As percentage of EI**																	
Total Fat (%)	–	51.6 ± 0.5	–	–	42.8 ± 0.5	–	–	30–40%	39.5 ± 0.5	10.8	–	46.4	30–40%	38.7 ± 0.4	12.0	–	43.5
Saturated fat (%) ^b^	–	20.8 ± 0.4	–	–	14.8 ± 0.4	–	–	–	11.9 ± 0.3	–	–	–	< 8%	13.2 ± 0.3	–	–	87.3
Carbohydrate (%)	–	42.1 ± 0.4	–	–	48.7 ± 0.5	–	–	45–65%	50.0 ± 0.5	27.5	–	5.9	45–65%	48.8 ± 0.5	34.1	–	3.8
Total Sugar (%)	–	40.0 ± 0.3	–	–	31.8 ± 0.6	–	–	–	25.6 ± 0.5	–	–	–	–	21.1 ± 0.4	–	–	–
Protein (%)	–	7.2 ± 0.1	–	–	9.4 ± 0.2	–	–	5–20%	11.5 ± 0.2	0	–	2.2	5–20%	13.8 ± 0.2	0.5	–	6.1

*Abbreviations*: *AI* Adequate Intake, *AMDR* Acceptable Macronutrient Distribution Range, *d* day, *DRI* Dietary Reference Intake, *EAR* Estimated Average Requirement, *EI* energy intake, *g* grams, *kcal* calories, *SE* standard error, *WHO* World Health Organization

^a^ DRIs in bold are EARs

^b^ WHO upper limit for saturated fat (8% of energy intake) for children above 2 years of age [32]

In 12–23.9 months old toddlers, the composition of the transitional diet from infancy to toddlerhood was characterized by lower fat and higher protein intake as %EI. In this age group, the intake of protein fell within the AMDR for almost all of the toddlers, with none of the children having protein intakes below the AMDR, and only 2.2% exceeding this range. Approximately 11 and 27% of the participating toddlers had fat and carbohydrate intakes below the AMDR. Conversely, almost half of toddlers (46.4%) exceeded the AMDR for total fat. Only 3.6% of children aged 12–23.9 months exceeded the AI level for dietary fiber. In preschoolers aged 24–47.9 months, protein intake fell within the AMDR for protein, with almost none of the children having intakes below this range, and a small proportion (6.1%) exceeding the AMDR. Approximately a third of the children (34.1%) had carbohydrate intakes below the AMDR, while more than 40% of the preschoolers exceeded the AMDR for total fat. In parallel, the majority of participating preschoolers (87.3%) exceeded the WHO upper limit for saturated fat (8% EI), while approximately half (48.6%) exceeded the AI level for alpha-linoleic acid. Only 11.5% of preschoolers exceeded the AI level for dietary fiber.

### Micronutrient intakes

The mean intakes of micronutrients (antioxidants, B vitamins and bone-related nutrients and other micronutrients) are shown in Table 4, by age group. In infants, (0–5.9 months and 6–11.9 months), mean intakes of micronutrients exceeded the AI for all vitamins and minerals under consideration, except for vitamin D in both age groups and magnesium in 6–11.9 months old infants. The proportions of infants with micronutrients’ intakes exceeding the AI were calculated. Amongst 0–5.9 months old infants, a high proportion exceeded the AI for all nutrients, except for vitamin D and magnesium (15.5 and 46.6% of infants had intake levels above the respective AIs). For infants aged 6–11.9 months, the proportions of children exceeding the AI were also high for most of the nutrients, except for vitamin D (21.6% had intakes above AI), magnesium (37.8%) and vitamin A (39.2%). Intakes of iron and zinc were inadequate (<EAR) in 45.3 and 21.6% of infants aged 6–11.9 months, respectively.

**Table 4:** Micronutrient intakes amongst Lebanese infants and young children, by age

Age group	0–5.9 months			6–11.9 months				12–23.9 months				24–47.9 months			
Nutrient	DRI	Mean ± SE	% > AI	DRI^**a**^	Mean ± SE	% < EAR	% > AI	DRI^a^	Mean ± SE	% < EAR	% > AI	DRI^a^	Mean ± SE	% < EAR	% > AI
	AI			AI/EAR				AI/EAR				AI/EAR			
**Antioxidants**															
Vitamin C (mg/d)	40	50.0 ± 1.6	94.2	50	60.1 ± 2.4	–	54.1	**13**	71.0 ± 3.3	5.4	–	**13**	75.2 ± 3.5	10.2	–
**B vitamins**															
Thiamin (mg/d)	0.2	0.4 ± 0.0	73.8	0.3	0.7 ± 0.0	–	85.1	**0.4**	1.0 ± 0.0	4.9	–	**0.4**	1.0 ± 0.0	5.1	–
Riboflavin (mg/d)	0.3	0.6 ± 0.0	99.1	0.4	0.9 ± 0.0	–	89.9	**0.4**	1.2 ± 0.0	2.2	–	**0.4**	1.3 ± 0.0	2.8	–
Niacin (mg/d)	2	4.1 ± 0.3	80.6	4	7.4 ± 0.4	–	82.4	**5**	11.2 ± 0.4	7.7	–	**5**	12.0 ± 0.4	11.7	–
Vitamin B-6 (mg/d)	0.1	0.2 ± 0.0	100.0	0.3	0.6 ± 0.0	–	85.1	**0.4**	0.8 ± 0.0	9.0	–	**0.4**	1.0 ± 0.0	7.6	–
Folate (μg dietary folate equivalents/d)	65	102.3 ± 6.3	61.2	80	172.2 ± 8.5	–	80.4	**120**	226.1 ± 11.1	20.3	–	**120**	199.1 ± 7.1	33.8	–
Vitamin B-12 (μg/d)	0.4	0.9 ± 0.1	100.0	0.5	1.4 ± 0.1	–	87.2	**0.7**	2.3 ± 0.3	10.8	–	**0.7**	2.8 ± 0.1	9.9	–
**Bone-related nutrients**															
Calcium (mg/d)	200	374.0 ± 13.9	98.1	260	525.4 ± 19.8	–	91.2	**500**	582.2 ± 18.7	44.6	–	**500**	715.6 ± 20.5	35.9	–
Phosphorus (mg/d)	100	193.4 ± 8.6	98.1	275	390.1 ± 16.5	–	68.9	**380**	566.4 ± 19.4	23.0	–	**380**	916.2 ± 19.6	5.3	–
Magnesium (mg/d)	30	32.5 ± 1.2	46.6	75	71.0 ± 3.2	–	37.8	**65**	122.6 ± 4.2	14.9	–	**65**	184.2 ± 4.1	2.8	–
Vitamin D (μg/d)	10	4.9 ± 0.4	15.5	10	6.4 ± 0.4	–	21.6	**10**	6.0 ± 0.3	84.7	–	**10**	4.2 ± 0.2	91.9	–
**Other micronutrients**															
Vitamin A (μg retinol activity equivalent/d)	400	519.9 ± 11.9	92.2	500	511.7 ± 17.2	–	39.2	**210**	532.5 ± 36.6	12.6	–	**210**	348.4 ± 23.3	35.1	–
Vitamin K (μg/d)	2	25.5 ± 2.2	100.0	2.5	42.1 ± 3.4	–	93.2	30	71.8 ± 6.8	–	78.8	30	67.1 ± 3.4	–	60.6
Iron (mg/d)	0.27	3.6 ± 0.3	79.6	**6.9**	7.8 ± 0.4	45.3	–	**3**	9.5 ± 0.3	9.0	–	**3**	8.1 ± 0.3	11.2	–
Zinc (mg/d)	2	2.9 ± 0.2	60.2	**2.5**	4.5 ± 0.2	21.6	–	**2.5**	5.9 ± 0.2	6.8	–	**2.5**	6.4 ± 0.2	4.1	–
Sodium (mg/d)^b^	110	152.9 ± 5.9	92.2	110/370	542.0 ± 33.1	–	67.6	800	1087.8 ± 45.8	–	60.4	800	1672.1 ± 42.8	–	88.6
Potassium (mg/d)^c^	400	557.7 ± 20.6	94.2	400/860	1024.3 ± 37.1	–	68.2	2000	1495.5 ± 48.0	–	19.4	2000	2126.1 ± 44.8	–	51.4

*Abbreviations*: *AI* Adequate Intake, *d* day, *DRI* Dietary Reference Intake, *EAR* Estimated Average Requirement, *mg* milligrams, *SE* standard error, *μg* micrograms

^a^ DRIs in bold are EARs

^b^ The AI for Sodium is 110 mg/d for 6–6.9 months and 370 mg/d for 7–11.9 months

^d^ The AI for Potassium is 400 mg/d for 6–6.9 months and 860 mg/d for 7–11.9 months

Amongst toddlers (12–23.9 months old), 84.7% of participating children had inadequate intakes of vitamin D (<EAR). Inadequate intakes of folate and calcium (<EAR) were also observed in 20.3 and 44.6% of toddlers, respectively. Similarly, only 19.4% of children aged 12–23.9 months exceeded the AI level for potassium. In preschoolers, the majority of children (91.9%) had inadequate intakes of vitamin D. In addition, inadequate intakes of folate, calcium and vitamin A were observed in approximately a third of children form this age group (<EAR). Potassium intake exceeded the AI in 51.4% of preschoolers.

Sodium intake was compared to its Chronic Disease Risk Reduction (CDRR) intake level (1200 mg for 1–3.9 year old children) [29], and the results showed that around 36 and 68% of children aged 12–23.9 months and 24–47.9 months had intakes exceeding the CDRR, respectively (data not shown).

## Discussion

This is the first study on dietary intakes of infants and young children in the EMR, and one of the few in this age group worldwide [33]. The study showed that milk and dairy products were the most important source of energy until the age of 2 years, after which an alarming increase in the consumption of sweets, sweetened beverages and desserts was noted. A low adherence to dietary recommendations was observed, particularly for fruits and vegetables. The study results showed that protein intake was within the recommendations for the vast majority of participating children. Although total fat intake was lower in toddlers and preschoolers compared to infants, yet more than 40% of toddlers and preschoolers exceeded the AMDR for fat and 87% of children aged 24–47.9 months exceeded the WHO upper limit for saturated fat. An alarming low intake of dietary fiber was observed in the large majority of participating children. The study has also identified several micronutrients inadequacies that ought to be targeted by future interventions.

### Energy intakes

Our findings showed that average dietary EIs exceeded the EER estimates in all age groups. Although visual aids were provided during the interview to assist in food portion estimation, an over-reporting of dietary intake may have occurred [34], given the unconscious inclination amongst parents to portray their child as eating well [34], and the difficulty to estimate food losses associated with spillage or spitting up [34]. Despite this potential for overestimation, it should be noted that the estimates of EI obtained in the present study were consistent with those reported in previous studies [35, 36]. The percent by which EIs surpassed the EERs was also consistent with that reported by Devaney et al. amongst US infants aged 6–12 months (123% in the US and 124% in Lebanon) and toddlers (131% in the US vs. 132% in Lebanon) [34]. In addition, and in agreement with our results whereby 65–75% of children from the various age groups had EI levels above the EER, a study conducted in Belgium showed that 63–76% of children aged 6–36 months exceeded the recommended daily intake level for energy [33]. It is possible that at least part of the observed discrepancy between EI and EER could reflect actual overconsumption of energy amongst Lebanese children. Anthropometric data provide some support for this suggestion, whereby 25.9% of participating infants and young children were found to be at risk of overweight, 9.2% were overweight and 3.4% were obese. Available evidence suggests that the prevalence of pediatric obesity has increased dramatically in Lebanon during the past two decades [14, 37]. Although obesity is multifactorial in etiology, it is well acknowledged that excessive EI may contribute to increased adiposity and weight gain amongst children [34].

### Food consumption patterns

Our study findings contributed towards the characterization of the transition in food consumption patterns across age, from infancy to the preschool years. It showed that, although the contribution of milk (and dairy) to EI was lower in older children compared to younger ones, it remained the major source of energy until the age of 2 years. Similar findings were described in the literature [33, 38]. For instance, the study by Huysentruyt et al. in Belgium showed that milk contribution to EI decreased from 41.3% in 6–12 months to 25.4% in 12–23.9 months, reaching 16.8% EI in 25–36 months old children [33]. In parallel, our study findings highlighted an alarming increase in the consumption of sweets, sweetened beverages and desserts with age, reaching 11.8% EI in toddlers and 18.5% EI in preschoolers. This high intake is of concern given its potential association with weight gain, metabolic abnormalities, and dental carries coupled with suboptimal micronutrient intakes and inadequate dietary diversity [39]. Available evidence also suggests that early life exposure to sugar in foods and beverages may lead to a life-time preference of sweet taste [39], and may influence SSB intake in later childhood as well as adolescence [39].

### Adherence to dietary recommendations

Our study also assessed adherence to dietary recommendations amongst children aged 1 year and above, given that food based dietary guidelines are still not available for younger infants in the US or in the EMR/Lebanon. Our findings showed that adherence to fruits and vegetable recommendations was low, ranging between 14.4 and 34.3% for fruits and between 17.8 and 20.7% for vegetables. Briefel et al. (2015) have also reported low adherence to fruit and vegetable recommendations amongst US preschoolers, with only 30% meeting the 5-a-day recommendation [40]. Insufficient fruit and vegetable consumption during early childhood may be associated with a higher risk of micronutrient deficiencies and respiratory illnesses in the short term [41, 42], while also increasing the risk of non-communicable diseases, later in adulthood [43–45]. In our study, only half of the participating children adhered to recommendations pertinent to milk and dairy products. Eldridge et al. (2019) showed that milk and milk products were amongst the food groups that predicted higher micronutrient adequacy in children aged 0–4 years [46]. A recent systematic review also provided evidence in support of dietary guidelines and the importance of regular consumption of dairy products amongst children to ensure or improve bone health [47]. This may be particularly important in countries that are undergoing the nutrition transition, with its characteristic lifestyle changes that favor the consumption of fast food and sweetened beverages, at the expense of milk [47].

### Macronutrient intakes

As observed in previous studies [35, 36], protein intake was found to be within the recommendations for the majority of children participating in the study [35, 36]. Fat intake was however high, with 46.4 and 43.5% of toddlers and preschoolers respectively exceeding the AMDR for total fat. The high intake of fat amongst Lebanese children contrasts with findings described in previous studies in the US [34–36], whereby FITS 2016 [36] showed that 7% of toddlers and 3% of preschoolers exceeded the AMDR for fat. Our study findings have also documented high intakes of saturated fat, with the majority of preschoolers exceeding the WHO upper limit of 8% of EI. High total fat and saturated fat intakes seem to be a hallmark of the Lebanese food consumption pattern with previous studies conducted amongst school-aged children, adolescents and adults reporting high intake levels of these nutrients [7, 10, 48]. The study findings highlight an issue of public health concern given the potential association of high fat and saturated fat intakes with unhealthy weight gain, obesity [49] and increased risk of cardiometabolic abnormalities in this age group [50]. A recent systematic review and meta-analysis of randomized controlled trials (RCTs) and prospective cohort studies emphasized that dietary guidelines for children should continue to recommend diets low in saturated fat [50]. The intake of dietary fiber was assessed and, in agreement with other studies’ findings [46], this intake was found to be low, with only 3.6% of toddlers and 11.5% of preschoolers meeting the AI level. These observations may be a reflection of the low intake of fruit and vegetables in the study sample. In addition, despite the fact that more than 60% of children met the dietary recommendations for grains consumption, it is important to note that grains were consumed in their refined, low fiber forms. In fact, whole grain consumption was very low, with only 6.2% of the study participants having reported its consumption (data not shown).

### Micronutrient intakes

Compared to our study findings in terms of micronutrient intakes, studies conducted in the US reported higher micronutrient intakes and adequacy in infants and young children. These differences may be due to that fact that, unlike studies conducted in the US [35, 36], our nutrient intake analysis did not include dietary supplements but was solely based on food sources. The observed differences may also reflect the discrepancies between the 2 countries with respect to food fortification practices, which are common in the US [51, 52] but quasi-absent in Lebanon. Alternatively, the results obtained in our study may reflect actual differences in food consumption patterns between Lebanese and US children, with the diet in Lebanon being lower in meat, fruits and vegetables, while being higher in added fats and oils as well as sweets and sweetened beverages [53]. Iron intake appeared to be an issue amongst infants aged 6–11.9 months, i.e. during the transitional diet with 45.3% of infants having intakes <EAR. This highlights an important issue for intervention in this age group, particularly that anemia in infants and young children continues to be a problem in Lebanon (Hwalla and Adra: Prevalence and selected determinant of iron deficiency anemia in women and under five children in Lebanon, unpublished) [54, 55]. Similarly, given the key role that zinc plays in normal growth, development and immune function, the subset of older infants (6–11.9 months) whose dietary intakes fell below the EAR of zinc (21.6%) should not be ignored. Only 37.8% of older infants exceeded the AI for magnesium, highlighting another potential concern with the composition of the transitional diet, given that magnesium plays an important role in regulating protein synthesis, nerve and muscle function, energy production and bone health [56]. In line with findings reported from the US [35, 36, 57], only a small proportion of Lebanese toddlers exceeded the AI for potassium (19.4%). In addition, low folate and low vitamin A intakes were observed amongst toddlers and preschoolers. Taken together, these findings may be a reflection of the low intake of fruits and vegetables in these age groups. Also common to both toddlers and preschoolers was a low dietary intake of calcium. Needless to say, the high proportion of toddlers and preschoolers not meeting the recommendation for milk and dairy products may explain the low intake of this nutrient. A low vitamin D intake was observed across all age groups, which is in line with findings reported amongst infants and young children living in France [58] and the UK [59]. Data on vitamin D deficiency amongst infants and young children do not exist in Lebanon, but available evidence suggests that estimates of vitamin D inadequacy from the diet are often higher compared to biochemical estimates from serum 25 (OH) vitamin D [36, 60].. The study has also investigated sodium intake and showed that around 36 and 68% of children aged 12–23.9 months and 24–47.9 months had intakes exceeding the CDRR Intake level [29], respectively. In agreement with our findings, a recent national survey of French infants and young children has also reported high sodium intakes in this age group [58]. The early establishment of taste preference for salt can affect food habits and choices later in life [61–63]. High sodium intake may also result in higher urinary calcium excretion [64], and impact blood pressure regulation [65, 66].

### Strengths and limitations

The strengths of this study comprise the national design of the survey and the measurements of anthropometric characteristics instead of self-reporting. The results of this study ought however, to be considered in light of the following limitations. In our study, dietary information was assessed based on the collection of a single 24-HR, which may not be representative of dietary intakes at the individual level. Future studies should consider the inclusion of a second 24-HR, at least for a sub-sample of the population. Despite the well-acknowledged limitations of the 24-HR approach, such as reliance on memory and possible day-to-day variation, the 24-HR may provide accurate estimates of EI at the population level [67]. In addition, dietary information was collected by the multiple pass approach, which was found to reduce the limitations of 24-HRs [68]. All recalls were administered by research nutritionists who went through extensive training prior to data collection in order to attenuate interviewer errors. Another limitation is the use of the USDA database in nutrient intake estimation, given the lack of food composition databases that are specific to Lebanon or even the region. The USDA database may not correctly capture the nutrient composition of local food varieties in Lebanon and may thus represent a source of error in intake estimations [69]. In addition, the USDA database does not include many of the composite traditional dishes consumed in the country. In order to address this limitation and be able to assess nutrient intakes from mixed traditional dishes, we have added standardized recipes to the Nutritionist Pro software using single food items [69]. In addition, micronutrient intake assessment was based on food and beverage intakes and did not take into consideration supplement use. Finally, given that the survey was conducted 10 years ago, its findings may underestimate the effects of the nutrition transition on dietary intakes in young children.

## Conclusions

In conclusion, this study highlighted an alarming high consumption of sweets, sweetened beverages, desserts and savory snacks especially among toddlers and preschoolers, a low adherence to recommendations pertinent to fruits and vegetables, a high intake of total fat, saturated fat and sodium, coupled with a low intake of dietary fiber in the study sample. The study has also identified several micronutrients of concern in the diets of infants and young children in Lebanon. Dietary strategies that foster the consumption of nutrient-dense foods and beverages at the expense of high fat, high sugar foods/beverages are needed. For instance, increased consumption of meats and fortified unsweetened cereals amongst 6–12 months old infants could increase iron intakes during this transitional diet period [46]. Increased consumption of fruit and vegetables could address nutrient shortfalls for potassium, folate, vitamin A and dietary fiber. Encouraging the adequate consumption of milk and milk products amongst toddlers and preschoolers will enhance the intake of calcium, while also contributing towards a decrease in the consumption of sweetened beverages. Concerted multi-stakeholder efforts that include parental education with specific actionable advice, professional training of medical and health practitioners, and government surveillance are needed to instill heathier food consumption and nutrient intake patterns early in life.

## Supplementary Information

**Additional file 1: Table A1**. Average mean intake (g/day) of the different food groups (as consumed) per capita, by age. **Table A2.** Average mean intake of the different food groups (after disaggregation of composite recipes), by age group.

## Data Availability

The datasets used and/or analyzed during the current study are available from the corresponding author on reasonable request.

## Abbreviations

24-HR: 24-hour dietary recall
AHA/AAP: American Heart Association/American Academy of Pediatrics
AI: Adequate Intake
AMDR: Acceptable Macronutrient Distribution Range
BMI: Body mass index
CDRR: Chronic Disease Risk Reduction
DRI: Dietary Reference Intake
EAR: Estimated Average Requirement
EER: Estimated energy requirements
EI: Energy intake
ELNAHL: Early Life Nutrition and Health in Lebanon
EMR: Eastern Mediterranean Region
FITS: Feeding Infants and Toddler Study
IOM: Institute of Medicine
LDL: Low-density lipoprotein
PAL: Levels of physical activity
RCTs: Randomized Controlled Trials
SE: Standard error
SSBs: Sugar-sweetened beverages
USDA: United States Department of Agriculture
WHO: World Health Organization
