# Supplementary material for: Food consumption patterns and nutrient intakes of infants and young children amidst the nutrition transition: the case of Lebanon
Source: Nutr J. 2022 May 23;21:34. doi: 10.1186/s12937-022-00779-9 (PMC9125916; doi:10.1186/s12937-022-00779-9)
Supplement: Supplementary file 1 — Additional file 1: Table A1. Average mean intake (g/day) of the different food groups (as consumed) per capita, by age. Table A2. Average mean intake of the different food groups (after disaggregation of composite recipes), by age group. [file 12937_2022_779_MOESM1_ESM.docx]

**Additional Material**

**Table A1. Average mean intake (g/day) of the different food groups (as consumed) per capita, by age**

| **Food Groups** | **Age group (months)** | | | |
| --- | --- | --- | --- | --- |
|  | **0-5.9 (n=103)** | **6-11.9 (n=148)** | **12-23.9 (n=222)** | **24-47.9 (n=393)** |
|  | **Mean ± SE** | | | |
| **Grain and grain products¹** | 5.59 ± 1.89 | 46.3 ± 5.01 | 85.17 ± 6.00 | 87.36 ± 4.43 |
| **Fruits²** | 4.61 ± 2.29 | 68.78 ± 8.42 | 106.2 ± 9.39 | 188.08 ± 10.49 |
| **Vegetables³** | 4.66 ± 4.66 | 25.16 ± 4.19 | 61.60 ± 6.60 | 101.10 ± 6.50 |
| **Milk and milk products⁴** | 472.23 ± 30.19 | 302.53 ± 20.75 | 170.06 ± 12.69 | 109.63 ± 6.67 |
| **Meats and other protein sources⁵** | 0.00 ± 0.00 | 6.85 ± 1.74 | 25.35 ± 3.21 | 59.89 ± 4.00 |
| **Mixed dishes⁶** | 0.89 ± 0.89 | 60.34 ± 8.20 | 131.87 ± 11.29 | 164.27 ± 9.25 |
| **Savory snacks⁷** | 0.00 ± 0.00 | 1.73 ± 0.53 | 7.18 ± 0.97 | 15.65 ± 1.40 |
| **Sweets, sweetened beverages, and desserts⁸** | 3.94 ± 2.94 | 69.91 ± 10.52 | 162.41 ± 13.35 | 255.8 ± 12.65 |
| **Fats and oils⁹** | 0.36 ± 0.27 | 8.77 ± 1.11 | 12.61 ± 1.06 | 14.62 ± 1.17 |
| **Condiments and sauces¹⁰** | 0.00 ± 0.00 | 0.02 ± 0.01 | 0.50 ± 0.22 | 2.29 ± 0.51 |

^1^ Includes bread, rolls, pita, saj, baby food cereals/grains, baby food finger food, cereals, crackers, pretzels, kaak, pancakes, French toast, pasta, rice, and other grains.
^2^ Includes baby food fruits, canned, dried, and raw fruits, 100% baby food juices, and other 100% fruit juices.
^3^ Includes baby food vegetables, canned, cooked and raw vegetables, white potatoes, and 100% vegetable juice.
^4^ Includes any milk (breast milk, infant formula, cow’s milk, and goat’s milk) as well as dairy foods, cheeses, and yogurt.

⁵ Includes any baby food and non-baby food meats, dried beans, peas and legumes, eggs, peanut butter, nuts and seeds.

^6^ Includes all yogurt, grain, and meat based mixed dishes such as sandwiches, macaroni and cheese, spaghetti and lasagna, sandwiches, beans and rice, pizzas, Mahashi, and soups.
^7^ Includes popcorn, potato chips, and corn chips.
^8^ Includes baby food desserts and cookies, non-baby food dessert items (cakes, pies, cookies, bars, brownies, biscuits, pastries, muffins, and traditional desserts), ice cream and dairy desserts, puddings, candy, cereal and nutrition bars, gelatins, ices, and sorbets, sugars, syrups, preserves, and jelly, fruit drinks and other sugar sweetened beverages.

⁹ Includes butter, margarine, animal fats, dressings, oils, and olives.

¹⁰ Includes condiments, herbs, seasonings, gravies, and sauces.

**Abbreviations:** g: grams; n: sample size; SE: standard error.

**Table A2. Average mean intake of the different food groups (after disaggregation of composite recipes), by age group**

| **Food group** | **Age group (months)** | |
| --- | --- | --- |
|  | **12-23.9 (n=222)** | **24-47.9 (n=393)** |
|  | **Mean ± SE** | |
| **Milk/dairy, cups** | 2.1 ± 0.1 | 2.3 ± 0.1 |
| **Lean meat/beans, grams** | 52.6 ± 5.5 | 94.7 ± 5.0 |
| **Fruits, grams** | 107.3 ± 9.4 | 190.9 ± 10.5 |
| **Vegetables, grams** | 102.7 ± 7.6 | 138.1 ± 6.4 |
| **Grains, grams** | 109.4 ± 5.9 | 124.8 ± 4.6 |

**Abbreviations:** n: sample size; SE: standard error.
